# Tryptophan Cluster Protects Human γD-Crystallin from Ultraviolet Radiation-Induced Photoaggregation *In Vitro*

**DOI:** 10.1111/php.12096

**Published:** 2013-06-20

**Authors:** Nathaniel Schafheimer, Jonathan King

**Affiliations:** Department of Biology, Massachusetts Institute of TechnologyCambridge, MA

## Abstract

Exposure to ultraviolet radiation (UVR) is a significant risk factor for age-related cataract, a disease of the human lens and the most prevalent cause of blindness in the world. Cataract pathology involves protein misfolding and aggregation of the primary proteins of the lens, the crystallins. Human γD-crystallin (HγD-Crys) is a major γ-crystallin in the nucleus of the human lens. We report here analysis of UVR-induced damage to HγD-Crys *in vitro*. Irradiation of solutions of recombinant HγD-Crys with UVA/UVB light produced a rise in solution turbidity due to polymerization of the monomeric crystallins into higher molecular weight aggregates. A significant fraction of this polymerized protein was covalently linked. Photoaggregation of HγD-Crys required oxygen and its rate was protein concentration and UVR dose dependent. To investigate the potential roles of individual tryptophan residues in photoaggregation, triple W:F mutants of HγD-Crys were irradiated. Surprisingly, despite reducing UVR absorbing capacity, multiple W:F HγD-Crys mutant proteins photoaggregated more quickly and extensively than wild type. The results reported here are consistent with previous studies that postulated that an energy transfer mechanism between the highly conserved pairs of tryptophan residues in HγD-Crys could be protective against UVR-induced photodamage.

## Introduction

Protein misfolding and aggregation are hallmarks of the pathology of many human diseases 1. Cataract is the leading cause of blindness in the world, projected to affect 20–30 million people in 2020, primarily the elderly, and is associated with misfolding and aggregation of the lens proteins 2. Despite the widespread prevalence of cataract, the relative contributions of identified risk factors to cataract have not been determined 3. One of the several risk factors identified is exposure to ultraviolet radiation (UVR).

Ultraviolet radiation is a ubiquitous environmental hazard for life on earth. Although the development of the ozone layer 2.4 billion years ago limited terrestrial UVR exposure to the UVA (400–315 nm) and UVB (315–280 nm) ranges, UVR still exerts selective pressure on extant creatures 4. DNA photodamage and its downstream impacts on the cellular level have been well studied 5–9. The accumulation of UVR-induced DNA lesions leads to mutations, the obstruction of DNA replication and, if unaddressed, cell death. Several DNA repair pathways have been identified and characterized that target UVR-induced DNA lesions.

Unlike DNA photodamage, most types of protein photodamage cannot be repaired by cell processes. In cells, damaged proteins can be poly-ubiquitinylated and degraded by the ubiquitin proteasome pathway or assembled into large aggresomes and disposed through autophagocytosis 10. In some specialized tissues, such as the lens, where cataract occurs, neither option is available 11–13.

The human lens focuses light onto the retina; to do so, it must remain translucent 12. As the epithelial cells in the lens terminally differentiate and produce large quantities of the crystallin proteins, they degrade their organelles and ribosomes 14. Due to the lack of protein turn over in the lens, the damaged and aggregated crystallins are not cleared from the lens fibers and accumulate as cataracts. Covalently damaged lens proteins have been shown to have decreased stability and solubility, and tend toward aggregation 15.

The three crystallin families, α, β and γ, comprise 90% of total lens protein and are present at *ca* 400 mg mL^−1^ 16. α-Crystallins are ATP-independent chaperones of the small heat shock protein family. These bind partially unfolded or damaged proteins, sequestering them, but cannot refold them 17,18. The β- and γ-crystallins are globular, two domain structural proteins of *ca* 20 kDa, related by sequence and structure homology 16,19. Each domain is composed of two Greek Key motifs and contains a number of highly conserved aromatic residues. The β-crystallins form oligomers through domain swapping; the γ-crystallins are monomeric 20.

Due to the lack of protein synthesis or degradation in the lens fiber cells, a model of cataract has been proposed in which aggregation-prone species accumulate over the lifetime of an individual and gradually titrate away free α-crystallin; when no free chaperone remains, aggregation occurs, causing cataract 21. HγD-Crys, the γ-crystallin chosen here for study, is extremely stable and one of the more abundant γ-crystallins in the human lens nucleus 22. Several mutations in the gene for HγD-Crys are known to be associated with congenital cataract 23,24.

Earlier studies have investigated how bovine lenses, extracted mixtures of bovine crystallins, and extracted mixtures of human crystallins have responded to photosensitizers and UVR, which generate reactive oxygen species (ROS) that can mediate photodamage to proteins 25–27. One photosensitizer studied, *N*-formylkynurenine, is similar to the tryptophan-based UV filters abundant in the lens 28. Extracted lenses and lens protein extracts grew cloudy when exposed to photosensitizers and UVR, with an increase in the insoluble protein fraction population and disruptions to crystallin structure. A rise in turbidity, cross-linked products, non-Trp fluorescence and the presence of ROS was also reported when mixtures of bovine and human crystallins were irradiated in the absence of photosensitizers 29. More recently, Estey *et al*. 30 showed that UVR causes nondisulfide cross-linking and nonnative aggregation in the corneal crystallin ALDH3A1.

Other studies found exposure to UVR caused cataract to develop in laboratory animals 31–33. When Ayala *et al*. 32 exposed rats to short bursts of 300 nm UVR, they observed the development of light scattering in exposed lenses in the weeks and months following irradiation. Other work has found that light scattering develops in guinea pig lens after UVA exposure *in vivo* in the laboratory 34. Further *in vivo* work by Giblin *et al*. 35,36 found that UVA and UVB blocking contact lenses prevented UVR-induced cataract in rabbits.

**Figure 1:**
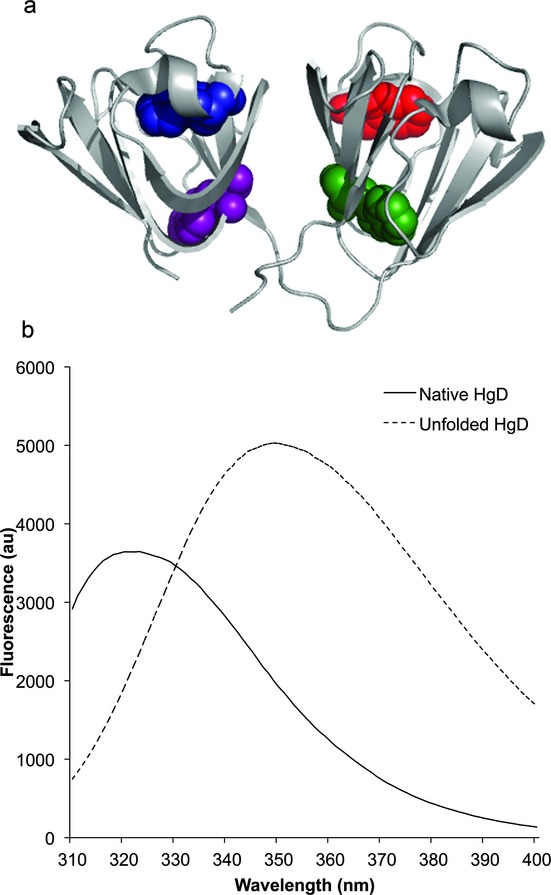
(a) X-ray crystallography structure of HγD-Crys 38 (PDB ID: 1HK0) displayed in ribbon form, with its four conserved Trp residues (W42 purple, W68 blue, W130 green and W156 red) highlighted in space filling form. (b) Graph of wild-type HγD-Crys Trp fluorescence emission spectra upon 295 nm excitation in the native (solid line) and GuHCl unfolded (dashed line) states.

The availability of high-resolution crystallin X-ray structures from Basak *et al*. enabled the discovery of a distinctive energy transfer mechanism at work in HγD-Crys between the conserved tryptophan pairs within the N- and C- terminal domains (Fig. 1a) 37,38. By examining fluorescence spectra and quantum yields of mutant HγD-Crys constructs, Chen *et al*. found evidence that one Trp of a pair (W68 or W156) has its fluorescence extremely quenched, whereas the other (W42 or W130 respectively) is moderately fluorescent and was shown to transfer its excited state energy to its quenched partner, resulting in anomalous native state Trp quenching (Fig. 1b). It was hypothesized that the mechanism could have evolved as a form of resistance to photodamage 39.

To investigate the molecular mechanism underlying photoaggregation of the crystallins, solutions of recombinant purified HγD-Crys were irradiated with a mixture of UVA/UVB, and the resulting photoaggregation monitored *via* solution turbidity, absorption spectroscopy, SDS-PAGE and transmission electron microscopy (TEM). Based on the fact that tryptophans absorb UVR the strongest and on the previously characterized energy transfer mechanism, we tested an initial hypothesis that photoaggregation proceeded through direct or indirect photodamage to one or more of the four Trp residues. Unexpectedly, we show here that damage to the Trp residues is unlikely to be on the pathway leading to the photoaggregated high molecular weight state. Rather the results reveal that the Trp residues may play a protective role.

## Materials and Methods

### 

#### Mutagenesis, expression and purification of HγD-Crys

N-terminally 6x-His tagged wild-type (WT) HγD-Crys expression constructs were modified *via* site-directed mutagenesis to introduce quadruple and triple W:F mutants 40. Constructs were confirmed *via* sequencing (Genewiz).

Recombinant WT HγD-Crys and mutant proteins were expressed and purified as described previously 40 with several modifications. Cells were grown to OD_600_ *ca* 1 in Super broth media at 37°C with shaking. Isopropyl β-D-1-thiogalactopyranoside was added to 1 mm and cultures were transferred to 18°C followed by shaking overnight. Cells were pelleted by centrifugation for 20 min at 17 000 ***g*** and resuspended in 30 mL Ni-NTA Lysis Buffer (300 mm NaCl, 50 mm NaPO_4_, 18 mm imidazole, pH 8) containing two tablets of Roche Complete EDTA-free protease inhibitor. After addition of lysozyme to 3 mg mL^−1^ and DNase to 3 μg mL^−1^, pellets were lysed *via* ultrasonication, and centrifuged at 17 000 ***g*** for 45 min. Supernatants were filtered and applied to a Ni-NTA column (GE Healthcare). Protein was eluted using a linear gradient of increasing imidazole concentration. Fractions containing the protein of interest were pooled and dialyzed three times against storage buffer (10 mm ammonium acetate, pH 7.0).

#### Protein concentration measurement

Stock protein sample concentration was determined using absorbance at 280 nm with the following extinction coefficients (determined using SIB's ProtParam): WT HγD-Crys 42 860 m^−1^ cm^−1^, triple W:F HγD-Crys 26 360 m^−1^ cm^−1^, quadruple W:F HγD-Crys 20 860 m^−1^ cm^−1^.

#### Photoaggregation experiments

Samples of HγD-Crys were prepared at 0.25 mg mL^−1^ or 1 mg mL^−1^ in 1x Reaction Buffer (100 mm Na_2_PO_4_, 1 mm EDTA, pH 7). Samples were irradiated at room temperature in a quartz cuvette (Starna Group) using a UVP Inc. UVLMS-38 lamp equipped with a 302 nm midrange bulb delivering a range of UVA/UVB light. UVR dose delivery was set to 2 mW cm^−2^, varied by adjusting the cuvette's distance to the lamp and determined before each experiment by a UVX Radiometer with midrange UVX-31 sensor. Turbidity readings at OD_600_ on a Cary UV/Vis Spectrometer were taken at regular time points during irradiation. Samples removed and analyzed *via* SDS-PAGE were reduced and boiled and electrophoresed through 14% acrylamide gels at 170 V for 1 h; gels were stained using Krypton Fluorescent Protein Stain (Thermo Fisher Scientific) and imaged on a Typhoon 9400 (Amersham Biosciences). Samples removed and analyzed using the bicinchoninic acid assay for protein concentration were filtered with a 0.2 μm membrane to remove aggregated protein and treated following the kit manufacturer's protocol (Thermo Scientific Pierce). The results were read on a Fluostar Optima plate reader (BMG Technologies). Aggregation rates were measured by calculating the steepest linear slope of the OD_600_ versus exposure time curve.

Oxygen-free irradiation experiments were conducted using a Coy anaerobic chamber under nitrogen. After an overnight incubation, samples were sealed into screw-top quartz cuvettes (Starna Group) with rubber stoppers before removal from the anaerobic chamber, and immediately used in photoaggregation experiments. An oxygen-sensitive dye solution (7.5 mm methyl viologen, 9 mm dithionite) in an identically sealed cuvette was used to confirm anaerobic conditions.

Action spectrum analysis of photoaggregation was achieved using small Newport Stabilife UVR cutoff filters to construct a shielding cage around the sample cuvette, and photoaggregation experiments were conducted as above.

#### Transmission electron microscopy

Five microliter samples of irradiated and unirradiated 0.1 mg mL^−1^ HγD-Crys sample in storage buffer were directly applied onto glow-discharged, carbon-coated, Formvar-filmed 400 mesh copper grids (Ted Pella). They were subsequently negatively stained with 1% uranyl acetate and blotted dry with filter paper. Sample grids were viewed in a transmission electron microscope (1200 XII; JEOL) and images were taken using an Advanced Microscopy Techniques XR41S side-mounted charge-coupled device camera.

#### Absorbance spectra measurements

Samples were collected from photoaggregation experiments and diluted into reaction buffer and 5 m guanidine hydrochloride (GuHCl) in black-walled tubes to minimize light scatter interference by aggregated protein. Samples were then incubated at 37°C for 6 h before scanning absorbance. Absorbance spectra of irradiated and unirradiated HγD-Crys samples were collected using a Cary UV/Vis Spectrometer.

#### Circular dichroism thermal unfolding measurements

CD spectra of the WT and mutant proteins were obtained using an AVIV model 202 CD spectrometer (Lakewood, NJ). Protein samples were prepared at a concentration of 0.1 mg mL^−1^ in 10 mm sodium phosphate, pH 7.0. Data were collected at 218 nm in a 1 cm quartz cuvette. Sample temperature was increased from 25 to 95°C in 1°C steps with 1 min of equilibration time per °C, followed by 5 s reads. Data were buffer corrected, and mean residue ellipticity was calculated. The mean residue ellipticity versus temperature data were fit to a sigmoidal curve using Kaleidagraph (Synergy Software), and the unfolding midpoints were calculated. The unfolding temperatures reported are averages of three thermal unfolding experiments.

## Results

### HγD-Crys photoaggregation under UVR

**Figure 2:**
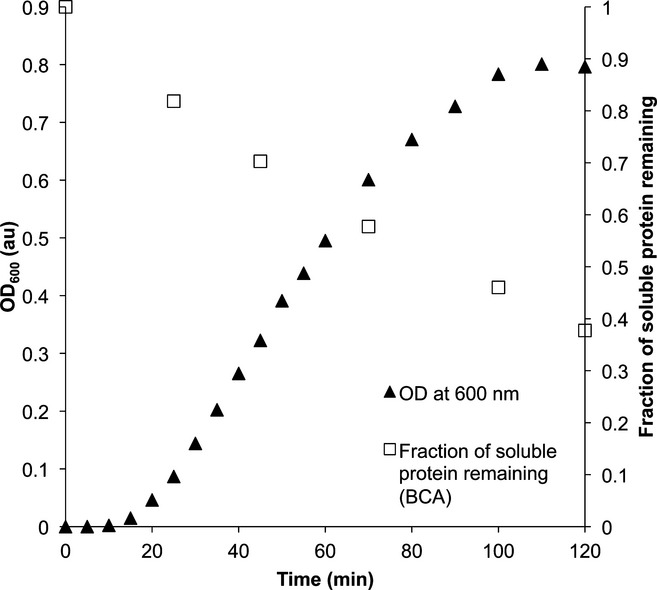
Changes in solution turbidity measured at OD_600_ (solid triangles, left axis) and changes in soluble protein concentration measured using the BCA (bicinchoninic acid) assay on samples (open squares, right axis) as a function of ultraviolet radiation exposure time (2 mW cm^−1^). Samples contained 0.25 mg mL^−1^ of wild-type HγD-Crys in sample buffer, and were incubated at 25°C.

We exposed purified HγD-Crys to UVR to investigate the underlying mechanism of photodamage in this highly stable lens protein. When WT HγD-Crys was exposed for 2 h to 2 mW cm^−2^ of UVR, solution turbidity rose dramatically after a lag period and then plateaued, consistent with previous studies and indicative of protein aggregation (Fig. 2). A lag period is often interpreted as evidence of a nucleation step in polymerization kinetics. However, we observed a shorter lag period when aggregation was monitored at 280 and 350 nm than those observed at 600 nm (Figure S1a, see Supporting Materials). This behavior suggests that the different lag times observed using different wavelengths of light represent detection of differently sized aggregates, and that, for the reaction under study, the apparent lag period is a consequence of initial aggregating species being too small to scatter light significantly.

As an additional approach to whether the lag phase represents a true nucleation step, we examined the lag time versus HγD-Crys concentration (Figure S1b, see Supporting Materials). Unlike well-documented nucleation reactions, the lag time was relatively insensitive to protein concentration 41.

At longer exposure times, the dose of UVR delivered to the sample may be lower than initially measured due to scattering in the sample. However, the OD_600_ continued to rise steadily even after the OD_280_ plateaued (Figure S1a), indicating continuing photoaggregation despite potentially lower dose delivered.

The concentration of WT HγD-Crys detected dropped steadily throughout UVR exposure to less than 40% its original concentration (Fig. 2), indicating more than 60% of the original sample's protein had entered an aggregated state by the time the OD_600_ had ceased increasing, but a significant population remained in solution.

### Photoaggregation dependencies

**Figure 3:**
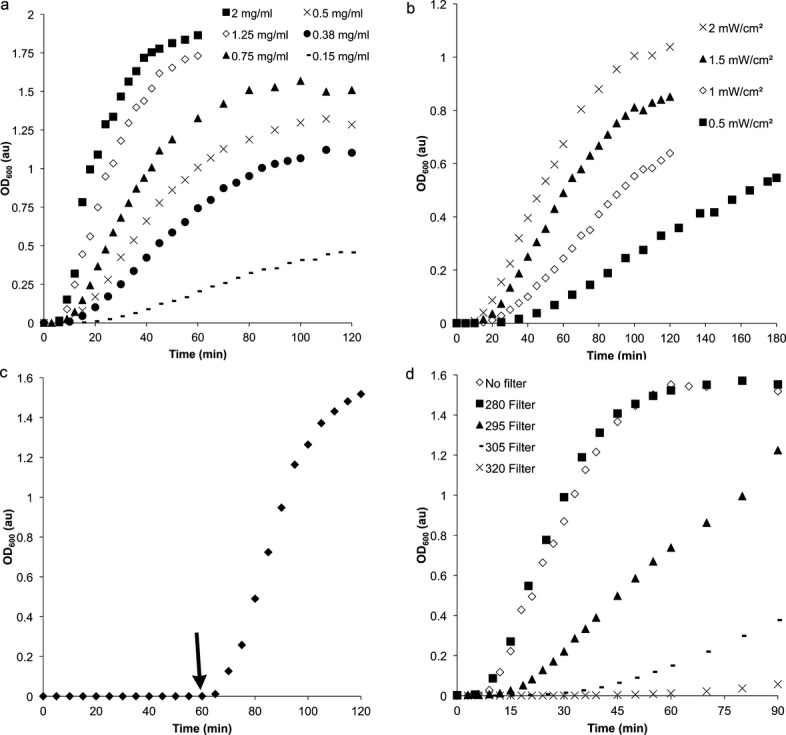
Parameters of ultraviolet radiation (UVR)-induced protein aggregation of wild-type (WT) HγD-Crys observed using OD_600_: (a) Irradiation of varying concentrations of WT HγD-Crys: 2 mg mL^−1^ (solid squares), 1.25 mg mL^−1^ (open diamonds), 0.75 mg mL^−1^ (solid triangles), 0.5 mg mL^−1^ (X's), 0.38 mg mL^−1^ (solid circles), 0.15 mg mL^−1^ (dashes). (b) UVR-induced aggregation of 0.25 mg mL^−1^ WT HγD-Crys samples using varying doses of UVR as measured *via* radiometer: 2 mW cm^−2^ (X's), 1.5 mW cm^−2^ (solid triangles), 1 mW cm^−2^ (open diamonds), 0.5 mW cm^−2^ (solid squares). (c) UVR exposure of a 1 mg mL^−1^ WT HγD-Crys sample in the absence or presence of atmospheric oxygen. Protein and buffer samples prepared anaerobically were irradiated, and then opened to the atmosphere at 60 min (denoted by arrow). (d) Exposure of 1 mg mL^−1^ WT HγD-Crys samples to decreasing ranges of the UV lamp's emission spectrum using glass filters blocking all light shorter than a wavelength cutoff: no filter (open diamonds), 280 nm filter (solid squares), 295 nm filter (solid triangles), 305 nm filter (dashes), 320 nm filter (X's).

To understand the parameters governing the photoaggregation of HγD-Crys, the irradiation and turbidity monitoring experiment was repeated, varying the concentration of WT HγD-Crys (Fig. 3a) and the dose of UVR (Fig. 3b). Increased rate of photoaggregation correlated with increased concentration of WT HγD-Crys and with UVR dose. The dependence of aggregation on temperature and pH was also examined. Photoaggregation did not show any clear dependence on either parameter (Figure S2 and S3, see Supporting Materials).

To determine whether oxygen played a role in the *in vitro* photoaggregation of HγD-Crys, buffer and protein samples were prepared anaerobically and then exposed to UVR doses as before (Fig. 3c). No change in turbidity was observed over an hour of UVR exposure under anaerobic conditions. At 60 min, oxygen was reintroduced to the reaction. As UVR exposure continued, solution turbidity developed robustly. These results indicated that oxygen is required to mediate photodamage for the *in vitro* photoaggregation of HγD-Crys.

Glass filters that sharply block all wavelengths shorter than specific thresholds were used to determine the action spectrum of photoaggregation (Fig. 3d). When wavelengths below 280 nm were blocked, there was no difference observed in the development of turbidity over exposure time. However, cutting off UVR at 295 nm and below, 305 nm and below and 320 nm and below progressively and dramatically slowed photoaggregation. This indicated an action spectrum encompassing the UVB range of *ca* 280–320 nm, but not the UVA range; this overlapped with the Tyr and Trp absorption spectra, as well as that of Trp photoproducts like kynurenine.

### HγD-Crys photoaggregate structure

**Figure 4:**
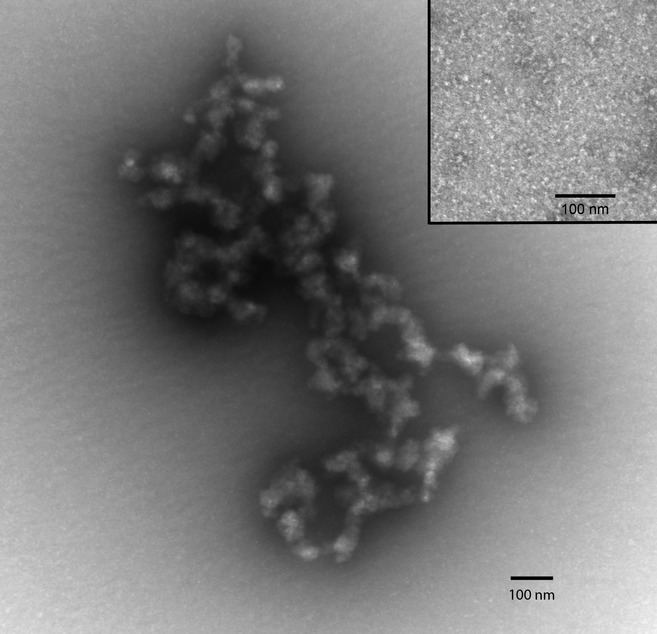
Transmission electron micrograph of uranyl-acetate stained ultraviolet radiation-induced aggregate from a 1 mg mL^−1^ wild-type (WT) HγD-Crys sample at 30 min irradiation at 2 mW cm^−2^. The inset is a negative control micrograph of unirradiated WT HγD-Crys.

Wild-type HγD-Crys photoaggregates were visualized using uranyl acetate negative stain TEM (Fig. 4). Aggregates observed were from 100 to *ca* 1000 nm in length. They were nonamyloid in structure but, while irregularly arranged, appear to have a rough, globular repeating unit *ca* 40–80 nm in size.

**Figure 5:**
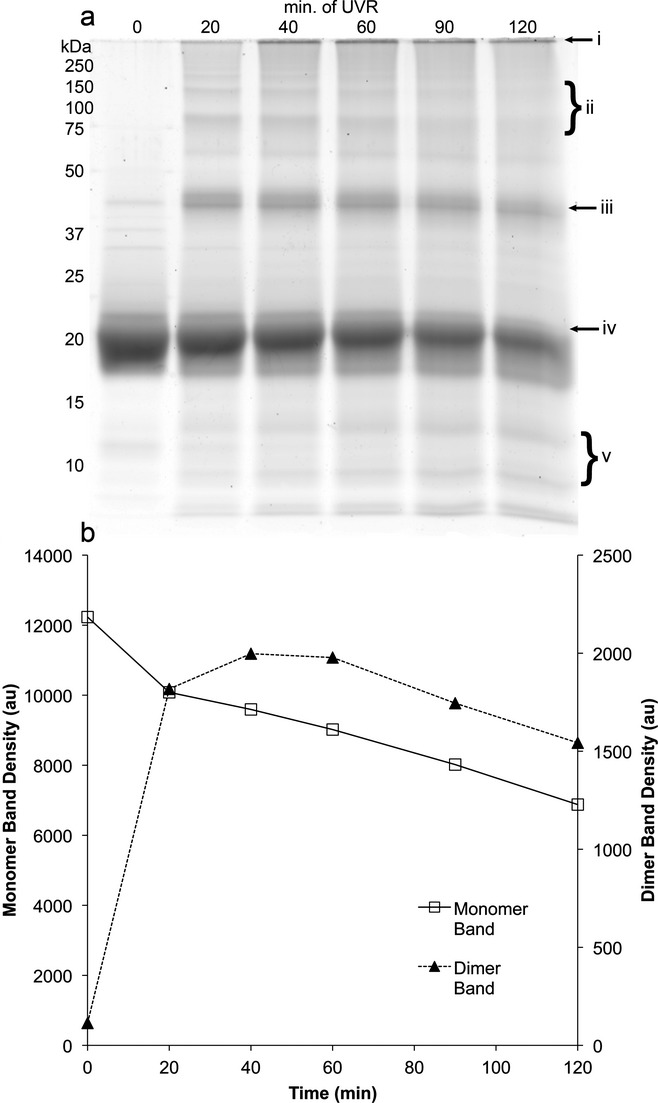
(a) Scanned image of a krypton-stained gel from SDS-PAGE of ultraviolet radiation (UVR)-induced aggregate samples from a 1 mg mL^−1^ wild-type HγD-Crys sample taken at a series of UVR exposure times: 0 min (lane 1), 25 min (lane 2), 45 min (lane 3), 70 min (lane 4), 100 min (lane 5), 120 min (lane 6). Marked sites: large protein aggregates unable to enter gel (i), high molecular weight species (ii), HγD-Crys dimer-sized band (iii), monomeric HγD-Crys band (iv), lower molecular weight degradation products (v). (b) Graphs of quantification of band density for the monomeric *ca* 20 kDa band (left axis) and the dimeric *ca* 40 kDa band (right axis) from the gel image in (a).

Samples of photoaggregation reactions of WT HγD-Crys were collected as a function of irradiation time and were analyzed by SDS-PAGE (Fig. 5a). All the samples exhibited a strong 20 kDa band, representing monomeric WT HγD-Crys (iv). After 25 min of UVR exposure, an *ca* 40 kDa band could be seen (iii) appropriate in size to be a HγD-Crys dimer. In addition, a series of high molecular weight bands (ii) appeared near the top of the gel, presumably multimeric species. By 45 min, a thin band at the top edge of the resolving gel could be seen, indicative of species too large to enter the gel (i). A series of lower molecular weight degradation products can be seen below the monomer band (v). Image analysis was used to quantify changes in band density between lanes, and showed that the monomer band diminished over time to half its original intensity over exposure time, while the dimer band increased until *ca* 45 min then diminished (Fig. 5b). This is consistent with the formation of an initial covalent dimeric cross-linked photoproduct that accumulates but is consumed by further photo–cross-linking and incorporated into larger aggregates.

**Figure 6:**
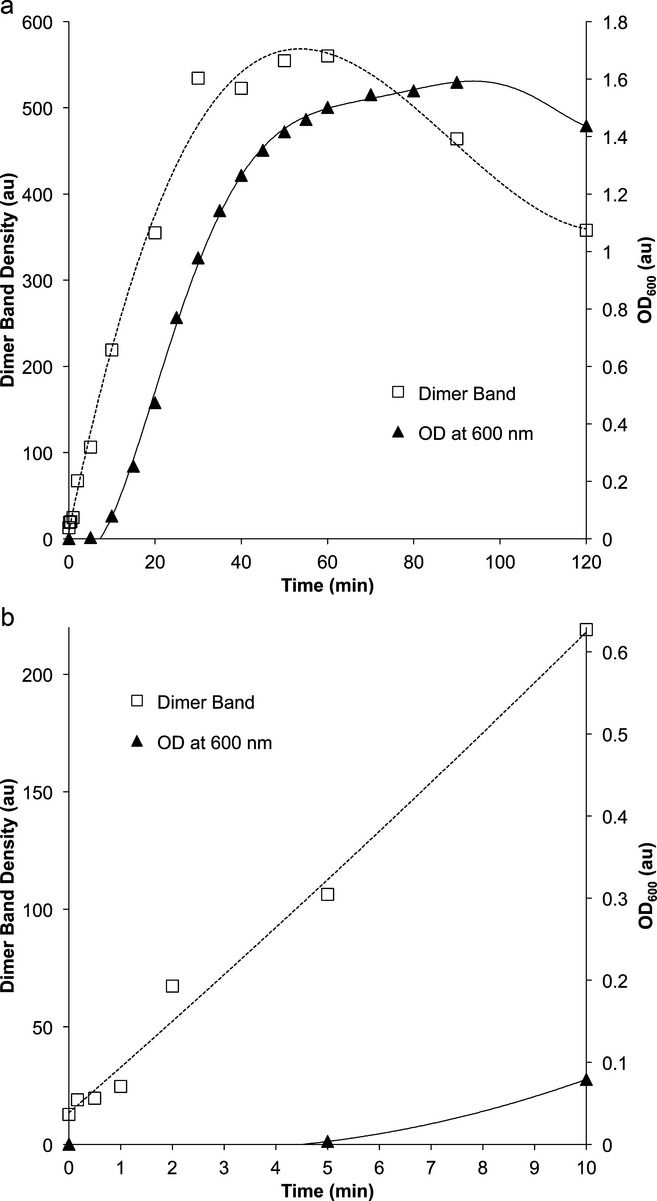
(a) Turbidity development at OD_600_ of 1 mg mL^−1^ wild-type (WT) HγD-Crys sample (black triangles and solid line, right axis) and dimer gel band density quantification from SDS-PAGE of the same WT HγD-Crys sample (open squares and dotted line, left axis) versus ultraviolet radiation (UVR) exposure time. Curves were generated using polynomial fits. (b) The same data are presented from (a), examining just the earliest time points from 0 to 10 min of UVR exposure.

When the density of the dimer band was examined at earlier UVR exposure times and compared with solution turbidity, we saw that the dimer appeared before OD_600_ increased, and began to wane as turbidity plateaued (Fig. 6a). At the earliest time points, with only 2 min of UVR exposure, the dimer band was detected (Fig. 6b). This suggests that the formation of a covalent dimer is an early step in the photoaggregation of HγD-Crys, and that solution turbidity measurements monitor the presence of later aggregation products.

### The role of aromatic residues in photoaggregation

**Figure 7:**
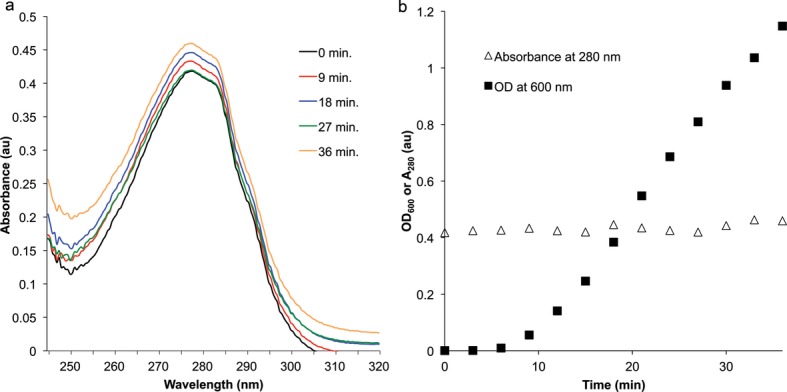
(a) Absorbance spectra of wild-type (WT) HγD-Crys at increasing ultraviolet radiation (UVR) exposure times, taken from 1 mg mL^−1^ samples diluted to 0.1 mg mL^−1^ into GuHCl to minimize aggregate light scattering: 0 min (black), 9 min (red), 18 min (blue), 27 min (green), 36 min (orange). (b) Graph of the change in solution turbidity at 600 nm (solid squares) and absorbance at 280 nm (open triangles) over UVR exposure time of the same WT HγD-Crys samples exposed to UVR as in (a).

We initially assumed that UVR absorption by tryptophans was a key step in photodamage, and that the photooxidized indole moiety was participating in free-radical polymerization. We therefore examined the absorbance spectra of HγD-Crys samples after varying UVR exposure times to assess whether photodamage occurred to aromatic residues (Fig. 7a). We observed no significant change in absorbance spectra in samples from 0 min to 36 min. However, over that same time period, aggregation proceeded robustly (Fig. 7b). The lack of significant changes in the absorbance spectra indicates an overall lack of damage to HγD-Crys's Trp's and Tyr's during photoaggregation.

To determine the role of Trp residues in photoaggregation, we examined triple and quadruple W:F mutant constructs of HγD-Crys, with a single Trp remaining or no Trp's remaining respectively. Previous studies established that these mutants folded into native-like structures 40. We confirmed that the mutant HγD-Crys proteins were stably folded by measuring far UV circular dichroism as a function of increasing temperature. Although all the mutant proteins were less stable than wild type, they retained their folded conformation to 60°C or above (Table 1).

**Table 1:** Circular dichroism thermal unfolding data for HγD-Crys mutant constructs

Construct	Melting temperature (°C)	Standard deviation (°C)
WT	82.1	0.4
W42 only	68.85	0.163
W68 only	67.46	0.0234
W130 only	65	0.146
W156 only	65.1	0.089
NoTrp	61	0.11

WT, wild-type.

**Figure 8:**
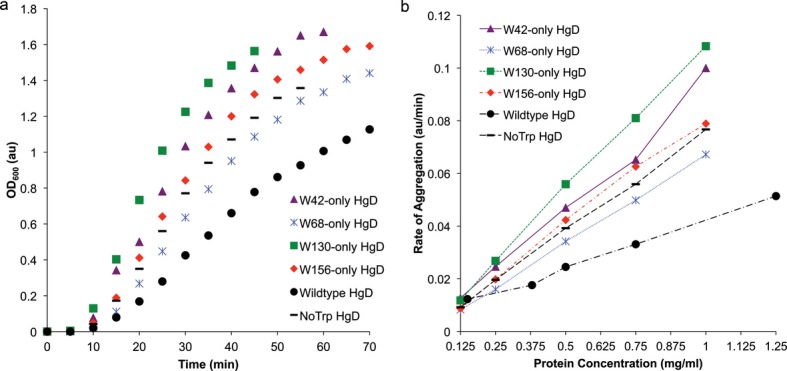
(a) Comparison of ultraviolet radiation (UVR)-induced aggregation of W:F mutant constructs of HγD-Crys by monitoring OD_600_ of 0.5 mg mL^−1^ protein solutions in sample buffer over irradiation time. (b) Comparison of the concentration dependences of UVR-induced aggregation of W:F mutant HγD-Crys constructs by analyzing the apparent rate of aggregation (steepest linear slope of OD_600_ curves) versus protein concentration.

In photoaggregation experiments with all four triple mutants and the NoTrp quadruple mutant, turbidity rose dramatically faster and reached a higher plateau than WT. This indicated that the W:F mutations made HγD-Crys more vulnerable to UVR-induced photoaggregation, not less, and that the absence of Trp residues did not retard photoaggregation (Fig. 8a).

We examined the concentration dependence of the rate of photoaggregation for WT and mutant HγD-Crys and found that the multiple W:F mutant HγD-Crys's diverge significantly in the concentration dependence of their photoaggregation rate (Fig. 8b). This implied that the W:F mutations significantly alter the photoaggregation pathway with respect to wild type.

## Discussion

The *γ*D-crystallins are very stable, resistant to denaturation both by chemical denaturants and heat, and are among the longer lived proteins in the human body 16. However, exposure to UVR *in vitro* results in rapid aggregation into high molecular weight complexes. As this reaction requires oxygen, it presumably involves photooxidation of certain residues.

HγD-Crys contains many aromatic amino acids capable of UVR absorption and radical photochemistry—four tryptophans, 14 tyrosines and six phenylalanines. Tryptophan has the highest specific absorption of protein amino acids at 280 nm, and HγD-Crys' 4 highly conserved Trp residues contribute 51.3% of its absorptivity, the remaining 48.7% coming from its 14 tyrosines 42,43. The Trp excited state can transfer its energy to other side chains or the peptide backbone, or scission of the indole ring of the excited residue can occur creating photoproducts, among them, kynurenine 44. In either path, absorption of UV photons by tryptophan could be an initial step in UVR-induced photodamage.

We monitored photoaggregation of HγD-Crys by two main methods, turbidity and SDS-PAGE. The inability to dissociate the aggregated state by boiling in SDS in the presence of reducing agents suggests covalent bonds between the subunits in the aggregated state. This suggests that aggregation of HγD-Crys was occurring *via* a radical polymerization mechanism 42.

SDS-PAGE revealed the formation of covalent dimeric photoproducts immediately after UVR exposure began. These subsequently decreased in intensity, consistent with a role as intermediates in the polymerization reaction. When aggregation was monitored by turbidity, an apparent lag phase was present. While a lag in an aggregation reaction could be indicative of a nucleation step in the aggregation mechanism 41, it was more likely a consequence of early aggregates being too small to scatter light at 600 nm; this is supported by the shorter lag times when turbidity was monitored at shorter wavelengths, as well as the absence of a lag in production of dimeric photoproducts.

The presence of distinct lower molecular weight bands of photoproducts suggests that photochemical scission of particular peptide bonds is also occurring. UVR-induced photochemical scission of peptide bonds 45 and degradation of crystallins 46 have been described previously. Although we cannot exclude the possibility that the fragments are incorporated into the covalent aggregated state, their steady increase during the course of UVR exposure is consistent with a photoproduct that is off the aggregation pathway.

We were surprised to find that in NoTrp HγD-Crys, the quadruple W:F mutant, photoaggregation occurred; this indicated that Trp was not necessary for UVR-induced aggregation, despite being the strongest UVR absorber in HγD-Crys. Counter-intuitively, removing half the UVR absorbing capacity of HγD-Crys actually increased the rate of photoaggregation. This suggests that the Trp residues, which are highly conserved among β/*γ*-crystallins 16, have a photoprotective role in HγD-Crys, and their replacement with Phe made HγD-Crys more vulnerable to photodamage. It also implicates the tyrosines of HγD-Crys, the remaining significant UVR absorbers, as playing a role in photoaggregation.

Such a photoprotective mechanism had been proposed to account for the super-quenched fluorescence emission found for the tryptophans of HγD-Crys as well as other crystallins 37. The human retina is very sensitive to UVR and the lens acts as a UV filter protecting the retina 47,48. Bova *et al*. 28 proposed that kynurenine and related metabolites served as UVR filters in the lens. The work of Chen *et al*. 49 makes the point that the β/*γ*-crystallins themselves—with their four conserved buried tryptophans—would also serve as UVR filters. The rapid quenching would then represent protection of the protein itself from UVR photodamage.

The occurrence of photoaggregation in the absence of Trp at first may appear inconsistent with several previous studies linking Trp photooxidative damage with the development of aggregation and/or cataract 50–53. Previous studies, however, often examined protein mixtures from human or animal lenses, or purified tryptophan in solution as opposed to a single recombinantly purified *γ*-crystallin protein. Such situations differ from the conditions under study here in their oxygen levels, the presence of other proteins and redox regulators. Other studies have also utilized photosensitizers to produce ROS and initiate oxidative damage to crystallins 26,27. UVR-induced damage has also been studied using laser radiation sources at differing wavelengths and sometimes of much higher power than the comparably physiological UVR dose being administered here 51,54. It is thus reasonable to expect a different photodamage pathway(s) to be encountered under the experimental conditions used here.

Interestingly, adding Trp's 42, 130 and 156 back (by examining triple W:F mutants with a single Trp remaining) slightly increased photoaggregation rates relative to NoTrp HγD-Crys, with W156 increasing the least and W130 the most, while adding W68 back slightly decreased the rate of photoaggregation. It would seem, overall, that having one Trp of the four is more deleterious for HγD-Crys photoaggregation vulnerability than having none at all. The photoprotective effect may require more than one Trp to be present, and there may be differences in the photodamage vulnerability and photoprotection contributed by the different Trp's.

The two triple W:F mutant HγD-Crys constructs with only a moderately fluorescent Trp remaining (W42 or W130) photoaggregated more rapidly than those with only a quenched Trp remaining (W68 or W156). This seems consistent with the analysis of Chen *et al*. 55, and suggests that the presence of a stronger fluorophore, and thus perhaps a longer lived photochemically active species, conveys stronger photoaggregation propensity than a weak fluorophore, which is photochemically active for a far shorter duration. Future experiments will address the effects of combinations of W:F mutations on photoaggregation.

An alternative explanation for the behavior of these multiple mutant HγD-Crys constructs is that, by modifying the hydrophobic core, the mutants proteins are destabilized or have taken on a nonnative conformation relative to WT. Assuming the mechanism of the observed photoaggregation involves an unfolding or partial unfolding step, these folding or structural changes would be responsible for the apparent photoaggregation rate change. However, previous experiments have characterized the multiple W:F mutant HγD-Crys constructs and found that they adopted stable, WT-like structures 40. CD thermal denaturation experiments showed that the mutants are, indeed, somewhat destabilized compared with WT (Table 1), but still unfold about 40°C above room temperature. Together, these suggest the changes in photoaggregation behavior result from phenomenon other than destabilization.

Another explanation for the differences in photoaggregation between WT and W:F mutants could be intraprotein cross-linking. If the conserved Trp residues become photoexcited and cross-linked to other sites within an individual HγD-Crys, such a reaction could compete with interprotein cross-linking and thus slow the observed aggregation with the formation of photoproducts with near identical molecular weights to monomeric HγD-Crys. Inserting W:F mutations would then be removing a competing chemical reaction pathway, not disrupting an energy transfer mechanism. However, this scenario is unlikely given the current data. Significant Trp-based intraprotein cross-links would cause a change in the absorbance spectra of the samples, which was not detected. Intramolecular cross-links, while not creating a significant difference in molecular weight between uncross-linked and cross-linked proteins, would also create small differences in their mobility in an SDS-PAGE gel, which were not seen.

From the current study, tryptophan is unlikely to be the site of photodamage, raising questions about which sites in HγD-Crys play key roles in UVR-induced photoaggregation. Excited state energy transfer occurs from tyrosine to tryptophan 44, and the current results suggest that one or a set of HγD-Crys' 14 tyrosines could be an important site of absorption and/or photodamage. Besides Trp, a number of amino acids, including Cys, Tyr and His, can participate in excited state radical chemistry that could lead to photochemical covalent cross-linking 56.

It seems likely that the mechanism of the observed photoaggregation involves absorption at a non-Trp aromatic site, the generation of a free radical on a residue of HγD-Crys, followed by several steps of covalent cross-linking to other HγD-Crys subunits. The absorption and reaction steps of this mechanism could proceed through an array of sites on the protein, or be very specific and involve a small number of residues. Preliminary analysis using mass spectrometry has not found evidence for a single dominant covalent cross-link, suggesting that cross-links are occurring at a diverse set of sites across HγD-Crys. The initial results have, however, identified significant oxidation of Cys18 in irradiated samples, consistent with previous reports on lens protein oxidation 57,58.

## SUPPORTING INFORMATION

**Figure S1.** (a) Changes in solution turbidity measuring OD_600_ (red triangles), OD_350_ (green diamonds) and OD_280_ (blue squares) over UVR exposure time. Samples contained 0.25 mg mL^−1^ WT HγD-Crys in sample buffer. (b) Lag time to turbidity development versus concentrations of WT HγD-Crys observed *via* OD_600_ (red triangles), OD_350_ (green diamonds) and OD_280_ (blue squares). Turbidity data were fit to sigmoidal curves and the time to increase to 0.05 au above baseline OD at time 0 was calculated as the lag time.

**Figure S2.** Changes in solution turbidity measuring OD_600_ of samples at pH 4 (blue diamonds), pH 5 (red squares), pH 6 (green triangles), pH 7 (purple X's), pH 8 (turquoise hatches) and pH 8.8 (orange circles) over UVR exposure time. Samples contained 0.25 mg mL^−1^ WT HγD-Crys in sample buffer. Solutions at pH 4 and 5 were produced by adding concentrated HCl to sample buffer beforehand. Solutions pH's were determined using a Thermo Orion PerpHect LogR Meter (model 330) with 8156BNUWP probe.

**Figure S3.** Changes in solution turbidity of 0.25 mg mL^−1^ WT HγD-Crys samples, measuring OD_600_, of samples at 4°C (blue diamonds), 16°C (red squares), 25°C (green triangles) and 37°C (purple X's) over UVR exposure time.
