# Supplementary figures and images for: Tryptophan Cluster Protects Human γD-Crystallin from Ultraviolet Radiation-Induced Photoaggregation In Vitro
Source: Photochem Photobiol. 2013 Jun 20;89(5):1106–15. doi: 10.1111/php.12096 (PMC3823069; doi:10.1111/php.12096)

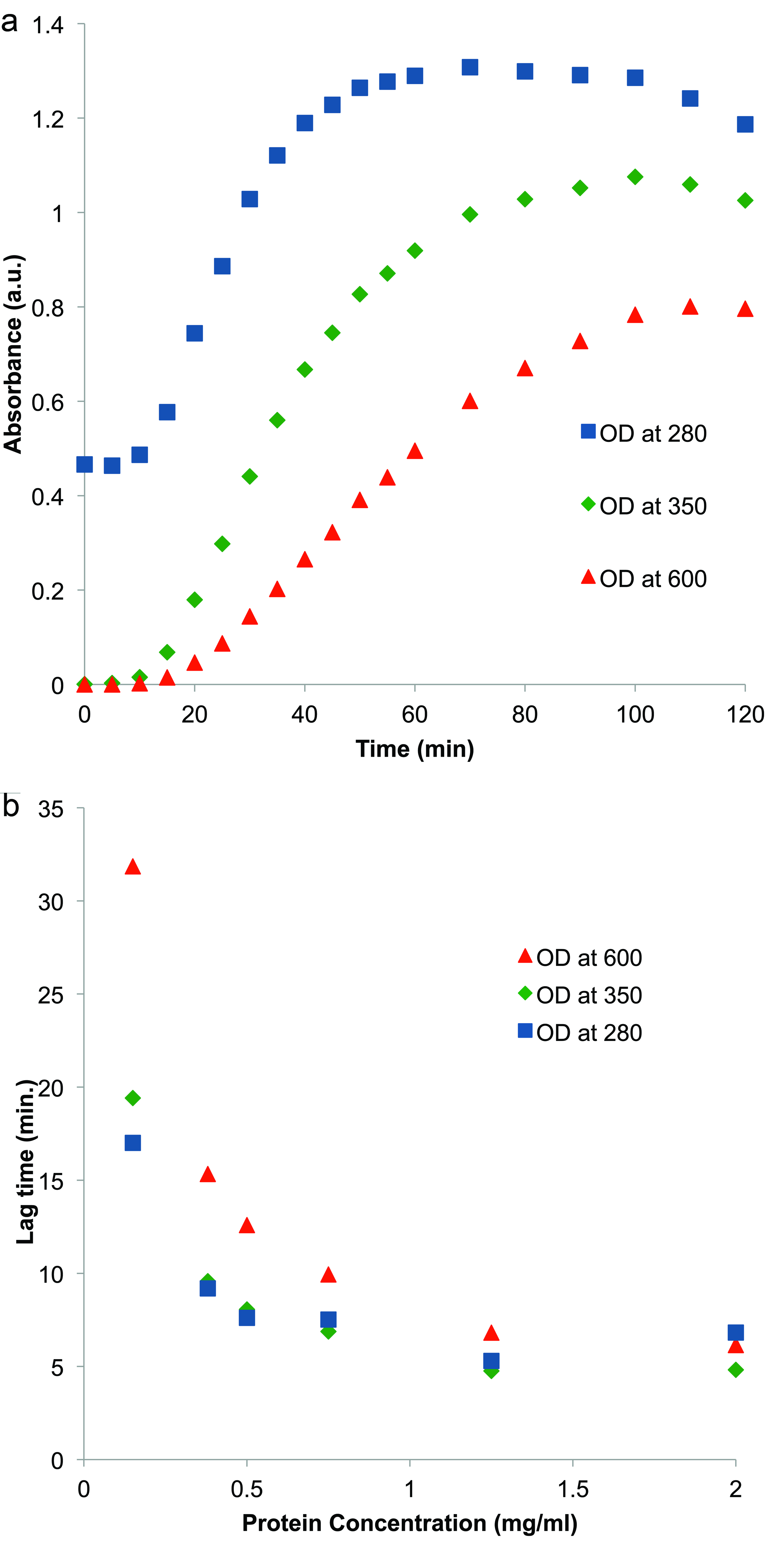

Supplement: Supplementary file 1 [file php0089-1106-SD1.tif]

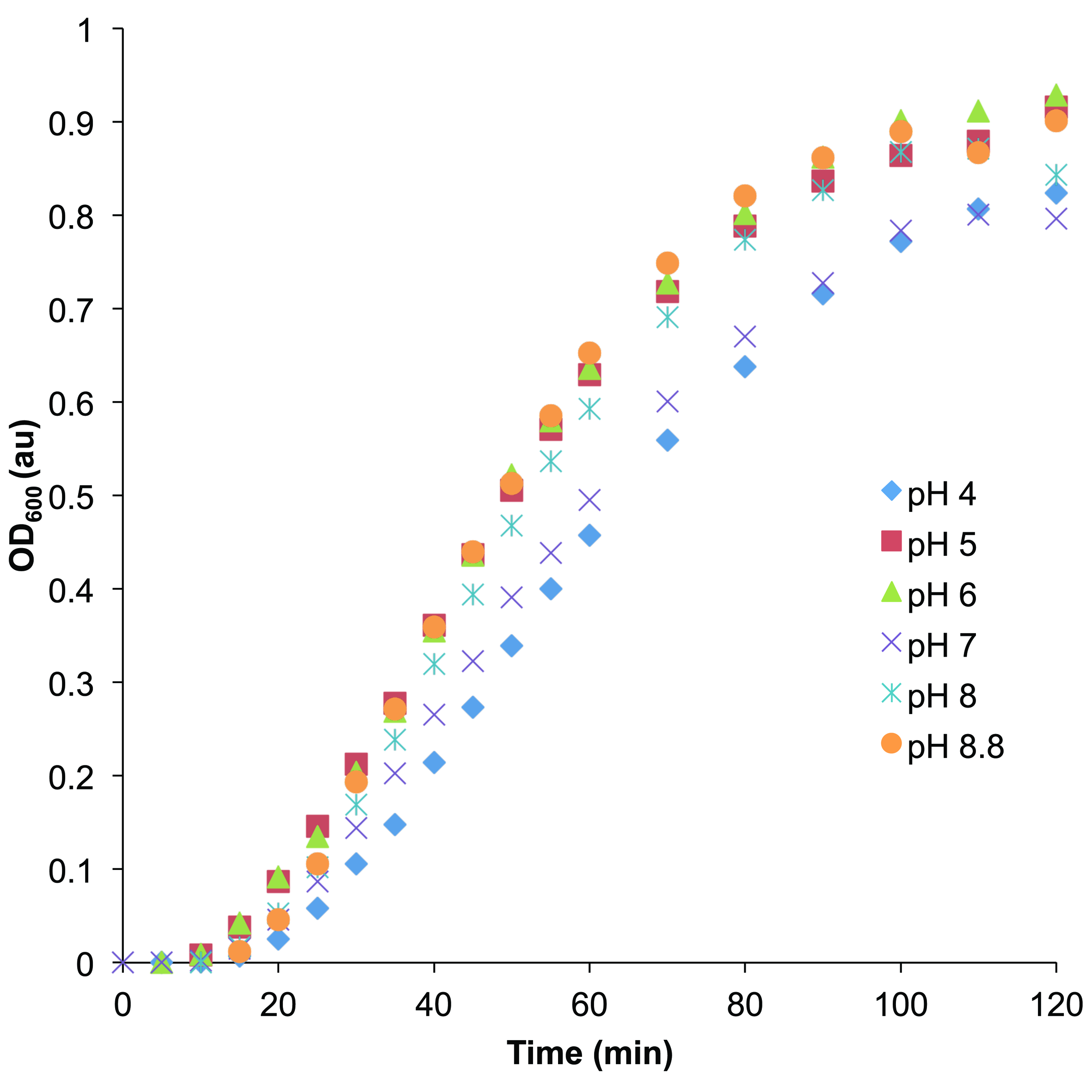

Supplement: Supplementary file 2 [file php0089-1106-SD2.tif]

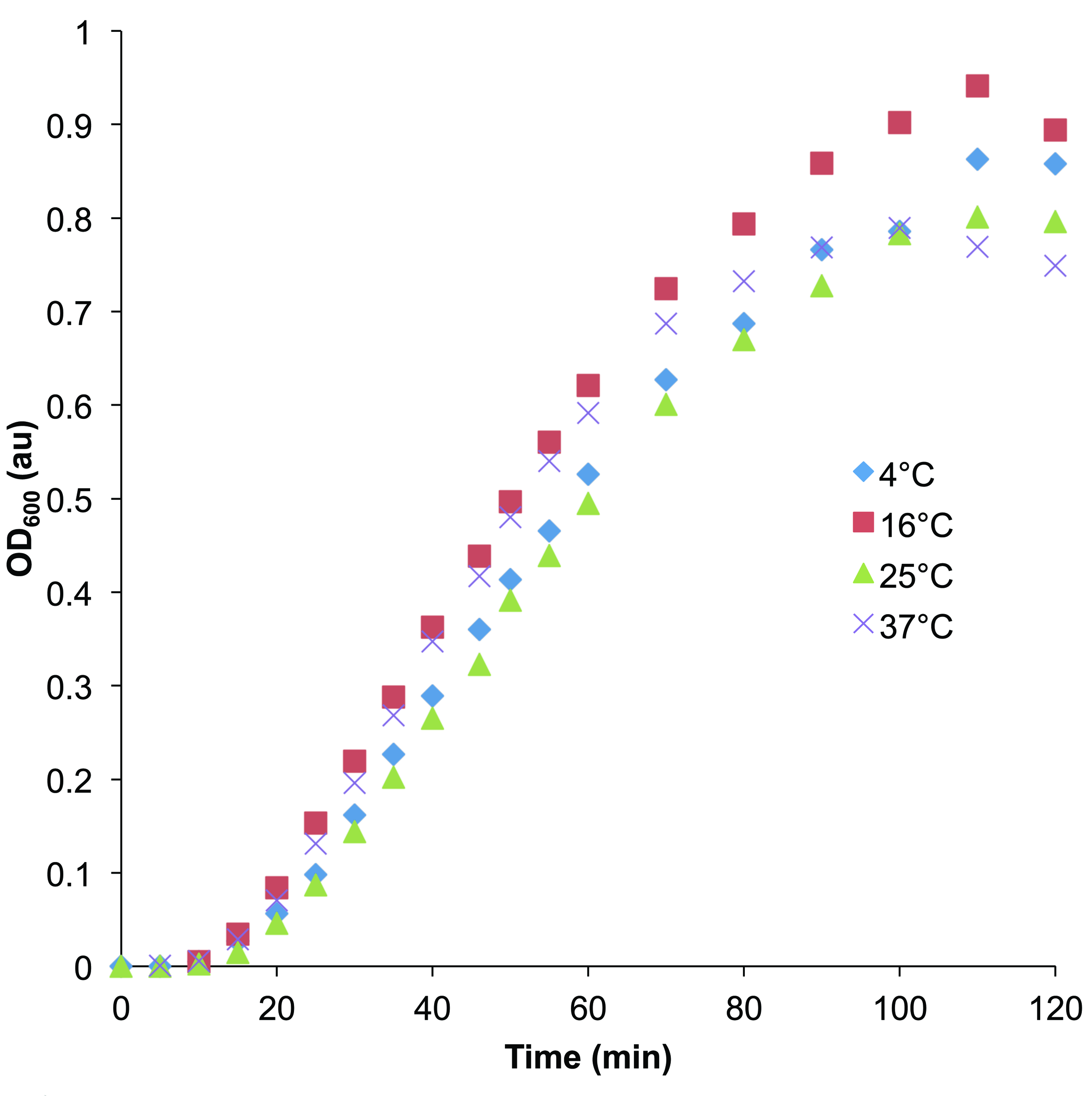

Supplement: Supplementary file 3 [file php0089-1106-SD3.tif]
